# Evaluation of the Orthotropic Behavior in an Auxetic Structure Based on a Novel Design Parameter of a Square Cell with Re-Entrant Struts

**DOI:** 10.3390/polym14204325

**Published:** 2022-10-14

**Authors:** Rodrigo Valle, Gonzalo Pincheira, Víctor Tuninetti, Cesar Garrido, Cecilia Treviño, Jorge Morales

**Affiliations:** 1 Faculty of Engineering, University of Talca, Talca 353 0000, Maule, Chile; 2 Department of Industrial Technologies, University of Talca, Talca 353 0000, Maule, Chile; 3 Department of Mechanical Engineering, Universidad de La Frontera, Temuco 478 0000, Araucania, Chile; 4 Department of Mechanical Engineering, University of the Bío-Bío, Concepción 403 0000, Bío Bío, Chile; 5 School of Engineering and Science, Tecnológico de Monterrey, Queretaro 76146, Mexico

**Keywords:** additive manufacturing, auxetic structures, cellular structures, mechanical characterization

## Abstract

In this research, a three-dimensional auxetic configuration based on a known re-entrant cell is proposed. The 3D auxetic cell is configured from a new design parameter that produces an internal rotation angle to its re-entrant elements to study elastic properties in its three orthogonal directions. Through a topological analysis using Timoshenko beam theory, the bending of its re-entrant struts is modeled as a function of the new design parameter to manipulate Poisson’s ratio and Young’s modulus. Experimental samples were fabricated using a fused filament fabrication system using ABS and subsequently tested under quasi-static compression and bending tests. Additionally, an orthotropy factor is applied that allows for measuring the deviation between the mechanical properties of each structure. The experimental results validate the theoretical design and show that this new unit cell can transmit an orthotropic mechanical behavior to the macrostructure. In addition, the proposed structure can provide a different bending stiffness behavior in up to three working directions, which allows the application under different conditions of external forces, such as a prosthetic ankle.

## 1. Introduction

Additive manufacturing (AM) gives us the opportunity to manufacture components with complex geometries at multiple scales and in different materials [1]. Challenging, conventional manufacturing methods to meet the high demands of engineering [2] have enabled the application of this technology in the manufacture of cellular structures [3], made up of nodes and struts rigidly interconnected through a periodic pattern [4,5]. The topology of these cellular structures is often inspired by nature [6,7,8,9,10], giving rise to the development of new materials whose microstructure exists in biological configurations [11,12]. These bioinspired structures are a promising solution for many engineering applications, depending on their characteristics. On the one hand, there are structures whose primary deformation is guided by tension, and these behave well in light structures due to their high stiffness and high strength. These can be applied in sandwich panels [13,14,15], which have been extensively studied in the literature [16,17,18]. One of the most studied structures, the honeycomb type [19], is used in the aerospace and packaging industry due to its high rigidity and low density [20,21]. Additionally, it is possible to find structures whose primary deformation is guided by bending; these structures have the ability to undergo large deformations under a relatively low stress level [8]. In addition, these structures have a high energy absorption capacity [22,23,24,25], making their application in protection structures possible.

These cellular structures can be designed with properties superior to traditional materials because their mechanical properties are not only defined by their chemical composition but also by their microscale topology [4,5]. This is why topology analysis becomes very important in designing the mechanical behavior of the structure based on its geometric parameters. Thus, the mechanical properties of cell structures can be potentially controllable since the unit cells can be tailored to meet particular requirements. Currently, there is extensive literature on the modeling and experimental characterization of this type of structure [26,27,28,29,30,31,32]. The benefits of 3D printing technologies have allowed researchers to study these types of periodical structures experimentally, both in two-dimensional panels and three-dimensional strut-based structures [33,34]. Some of the most studied designs are those dominated by tension through periodic panels, which may experience higher yield strengths than designs dominated by flexion [33,34,35]. This is because the intersections between plates ensure that the topology is always dominated by tension in any load direction, offering great structural stability [22,31]. However, this implies an increase in relative density since the structure is made up of panels with a larger section than a strut. On the other hand, one of the cellular structures that has attracted great attention is the auxetic structure. Unlike other structures, these contract laterally when subjected to compression and expand laterally when subjected to tension. This produces a negative Poisson’s ratio and is called the auxetic effect [36]. Thanks to this effect, these materials can experience various attractive properties such as high strength [37], high shear stiffness [38], great fracture toughness [23,39], and a remarkable energy absorption capacity [8,34]. Some more promising auxetic configurations are studied in [33,40]. Where the mechanical behavior of partially auxetic and totally auxetic structures is evaluated. The latter has a negative Poisson ratio in all its working directions. Therefore, it is accepted in the scientific nomenclature as simply Auxetic [41,42]. However, the most studied is the structure with re-entrant struts. This structure stands out mainly for its great capacity to absorb impact energy thanks to the primary deformation of the structure being guided by the bending of its re-entrant elements [33]. This has allowed dynamic studies, as in [43], experimental and analytical analysis was carried out to determine its mechanical behavior under cyclic loads. However, studies of these re-entrant structures date back to the pioneering work of Almgren 1985 [44], Lakes 1987 [45], Wojciechowski 1987 [46], Wojciechowski 1989 [47], and Evans 1989 [48], where the field of auxetic materials begins, proposing the first negative Poisson’s ratio structures, where microstructures that exhibit transverse expansions under longitudinal loading were modeled. Until more recent research, in [38,49], where the design of the structure was detailed to manipulate the auxetic effect of the cell. It was found that the more negative Poisson’s ratio, the more resistant the structure to shear deformation. Later, in [50,51], an analytical model was established using the classical theory of Timoshenko beams, based on a unit cell analysis to manipulate Poisson’s ratio, Young’s modulus, and the elastic limit of the cell structure. Given the good agreement between theory and experimentation, in [33], the same model was applied to analyze different auxetic cell configurations to adapt Poisson’s ratio and Young’s modulus. Theoretical, numerical, and experimental analyses were carried out to reveal the mechanical properties of different cell structures. Furthermore, the field of auxetic structures is not limited only to lattice structures but also to compounds whose topology is silvered at the atomic level [52,53,54], which also strongly influences the mechanical properties of the macrostructure. Thus, the literature shows theoretical analyses and experimental characterization to explore the mechanical properties of auxetic cell structures.

Anisotropy, orthotropy, and tension–compression asymmetry for bulk materials are well-studied phenomena in materials science and engineering design [55,56,57,58,59,60]. In addition, in [42], a relationship between totally isotropic to strongly anisotropic elastic properties with non-auxetic, partially auxetic, and auxetic behaviors is observed. On the other hand, materials obtained by additive technology and the orientation factor introduce intrinsic anisotropy, obtaining cellular structures with anisotropic mechanical properties without modifying their topology. Material anisotropy, as well as cell topology design, are important design factors [61,62]. In [63], an analytical model was applied to evaluate the effects of topology in a re-entrant auxetic cell structure considering the anisotropy factor of the material. Although the anisotropic properties of cell structures induced by manufacturing processes have been studied, there are no studies where the cell topology is analyzed to obtain different mechanical properties for each working direction. Therefore, most of the structures reported in the literature are designed to have homogeneous mechanical behavior in their three orthogonal directions and could only experience orthotropic behavior by modifying their macroscale geometry. This implies a higher computational cost to determine the mechanical behavior that the structure will have. This article presents the theoretical and experimental analyses to develop a new auxetic structure with re-entrant struts with different mechanical properties in its three orthogonal directions. Through the classical Timoshenko beam theory, the bending of the re-entrant elements is modeled to control Poisson’s ratio and Young’s modulus. Additionally, an orthotropy factor is applied that allows for measuring the deviation between the mechanical properties of each structure. This allows the development of a unit cell with an orthotropic mechanical behavior to build a macrostructure with an adaptable mechanical response to different external conditions.

This article is organized as follows: Section 2 presents the description of the theoretical model of Timoshenko to design the geometry of the 3D structure to obtain an orthotropic behavior, as well as the experimental procedure for the mechanical characterization. Finally, Section 4 presents the conclusions and future work.

## 2. Materials and Methods

### 2.1. Orthotropic Mechanical Model of the Cell

**Figure 1:**
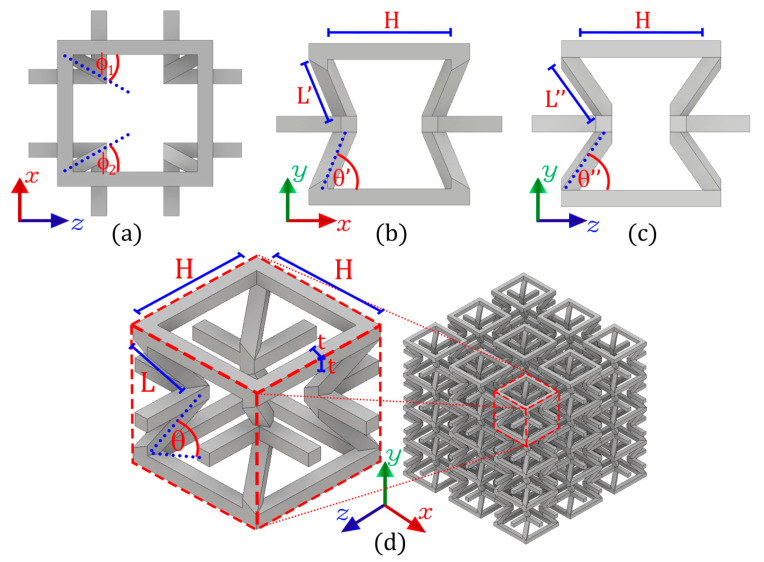
Auxetic structure design with re-entrant elements oriented under an angle of rotation ∅ (**a**). This modification in the topology produces two different transverse compression planes: (**b**) and (**c**). This unit cell can be modeled into a macrostructure, ensuring connectivity between cells (**d**).

Thimoshenko’s classical beam theory has been shown to predict with great success the mechanical properties of auxetic structures with re-entrant struts [33,38,49,53,54]. This analysis of the classic 2D auxetic cell with re-entrant struts is used as the basis for the design of a new structure with orthotropic behavior. This design includes a new design parameter ∅ that produces a rotation angle in the re-entrant elements of the Cell (0<∅<45°), as Figure 1a shows. This produces a difference in the orientation of the re-entrant elements for the planes shown in Figure 1b,c. In this way, we use the Timoshenko beam theory to model the bending of the re-entrant elements to manipulate the mechanical behavior of this new cell in its three orthogonal directions. This model is developed based on the 5 design parameters of the cell: the vertical length H, the length of the re-entering struts L, the re-entrant angle θ, the thickness of the cross-section t, and the new parameter ∅ that allows the re-entrant elements of the structure to rotate. It should be noted that $L^{\prime }$, $L^{\prime \prime }$, $\theta^{\prime },$ and $\theta^{\prime \prime }$ can be geometrically calculated based on the design parameters defined above. It is worth mentioning that the asymmetry of this new 3D cell does not prevent connectivity between neighboring cells from forming a macrostructure, as shown in Figure 1d.

**Figure 2:**
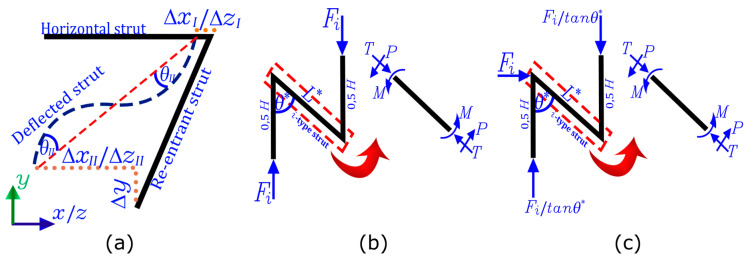
Simplified analysis of the auxetic structure, where (**a**) corresponds to the deformations of each re-entrant strut; (**b**) shows the distribution of the internal loads under compression on the y axis; while (**c**) shows the distribution of the internal loads under compression on the x and z axes.

As is known, the deformation of auxetic structures with re-entrant struts is produced by the elastic bending of their struts. Therefore, it is possible to model its mechanical behavior through the classical Timoshenko beam theory [33,38,49,53], according to the decomposition shown in Figure 2. However, it has been shown in previous works [64,65] that Timoshenko’s simplified analysis successfully predicts the mechanical behavior of an auxetic structure with an asymmetric design. Therefore, neither the shear strain Δy nor the axial strain of the horizontal elements $\Delta x_{I}$ and $\Delta z_{I}$ will be considered for this analysis since they are considered negligible.

Therefore, the simplified Timoshenko analysis considers only the bending of the re-entrant struts, which are modeled as a cantilever beam, to then determine their internal loads depending on the direction in which the structure is compressed, according to the decomposition shown in Figure 2. Thus, the simplified form is described by Equation (1). For more details, the reader is referred to [64].

(1) $$\theta_{II}=\frac{ML}{6E_{s}I}$$

where $E_{s}$ is Young’s modulus, I is the moment of inertia of the cross-section ($t^{4}$ for a t×t section), M represents the bending moment, and L is the length of the re-entrant strut.

#### 2.1.1. x Axis Compression

As shown in Figure 1, when compression is applied in the x axis direction, loads are transmitted onto the structure through the plane shown in Figure 1b. Therefore, considering that each re-entrant strut is an element shared by two adjacent cells, the compression force acting on each strut is:

(2) $$F=\frac{\sigma }{2}\;H\;L\;sin\theta \;$$

where *σ* is the compression stress. Once the compression force on each strut has been calculated, it is possible to calculate the internal loads T, P,  and M to later calculate their deformations. Due to the symmetry of cell 2 (b) struts will suffer the same deformations. Therefore, Poisson’s $v_{yx}$ ratio can be estimated based on the geometry as:

(3) $$v_{yx}=-\frac{\varepsilon_{y}}{\varepsilon_{x}}=-\frac{\left(\Delta y_{I}+\Delta y_{II}\right)L\;sin\theta \;\;}{\Delta x\left(H-L\;cos\theta \;\right)}=-\frac{sin^{2}\theta {}^{\prime }}{\;cos\;\theta {}^{\prime }\left(\frac{H}{L{}^{\prime }}-cos\theta {}^{\prime }\right)\;}$$

where $\varepsilon_{y}$ and $\varepsilon_{x}$ correspond to the strains in the x and y direction, respectively. Similarly, Young’s modulus $E_{x}$ can be determined as:

(4) $$E_{x}=\frac{\sigma }{\varepsilon_{x}}=\frac{\sigma t^{4}\left(\frac{H}{L{}^{\prime }}-cos\theta {}^{\prime }\right)}{F{\left(L{}^{\prime }\right)}^{2}sin^{2}\theta {}^{\prime }}\;E_{s}$$

#### 2.1.2. z Axis Compression

When compression is applied in the *z* direction, loads are transmitted to the structure through the plane shown in Figure 1c. Therefore, according to the tributary area, the compression force acting on each strut is:

(5) $$F=\frac{\sigma }{2}\;H\;L\;sin\theta \;$$

where σ is the compression stress. The internal actions of each strut T, P, and M are shown in Figure 2b. Therefore, Poisson’s $v_{yz}$ ratio can be estimated for each strut as:

(6) $$v_{yx}=-\frac{sin^{2}\theta^{\prime \prime }}{cos\theta^{\prime \prime }\left(\frac{H}{L^{\prime \prime }}-cos\theta^{\prime \prime }\right)}$$

Similarly, Young’s modulus $E_{z}$ can be determined as:

(7) $$E_{z}=\frac{\sigma t^{4}\left(\frac{H}{L{}^{\prime \prime }}-cos\theta {}^{\prime \prime }\right)}{F{\left(L{}^{\prime \prime }\right)}^{2}sin^{2}\theta {}^{\prime \prime }}\;E_{s}$$

#### 2.1.3. y Axis Compression

When compression is applied in the y direction, the geometric configuration shown in Figure 1a becomes the support structure. Unlike the compression directions x and z, in this case, the rotation of the re-entrant elements ∅ has no influence on the bending of these elements. However, it only has an influence on the transverse deformations of the cell. In this way, the compression force acting on each element is:

(8) $$F=\frac{\sigma }{4}\;H^{2}$$

where *σ* is the compression stress. The internal actions of each strut T, P, and M are shown in Figure 2c. Similarly, the Poisson ratios $v_{xy}$ and $v_{zy}$ can be estimated by analyzing the deformations for each strut, such as:

(9) $$v_{xy}=-\frac{cos\theta {}^{\prime }\left(\frac{H}{L{}^{\prime }}-cos\theta {}^{\prime }\right)}{sin^{2}\theta^{\prime }\;}$$

(10) $$v_{zy}=-\frac{cos\theta {}^{\prime \prime }\left(\frac{H}{L{}^{\prime \prime }}-cos\theta {}^{\prime \prime }\right)\;}{sin^{2}\theta^{\prime \prime }\;}$$

Finally, Young’s modulus $E_{y}$ can be determined as:

(11) $$E_{y}=\frac{\sigma t^{4}\left(\frac{H}{L}-cos\theta \;\right)}{F\;L^{2}\;sin^{2}\theta \;}E_{s}$$

#### 2.1.4. Poisson’s Ratio and Young’s Modulus

**Figure 3:**
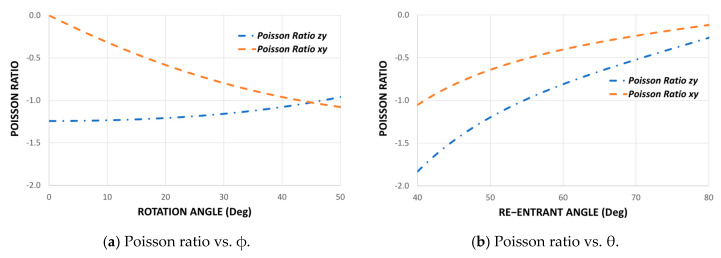
Average Poisson’s ratio for different values of design parameters θ and ∅, considering H=13.6 mm, L=5.9 mm and $\emptyset =\emptyset_{1}=\emptyset_{2}=22.5{}^{\circ}$.

Using the design parameters H, L, t, θ, and ∅, it is possible to demonstrate the elastic behavior of this new auxetic cell in its three working directions. As shown in the previous section, the beam theory allows obtaining the deformations of each re-entrant strut to establish the Poisson’s ratio through Equations (3), (6), (9), and (10). Figure 3 shows Poisson’s ratio as a function of the re-entrant angle θ and the angle of rotation.

**Figure 4:**
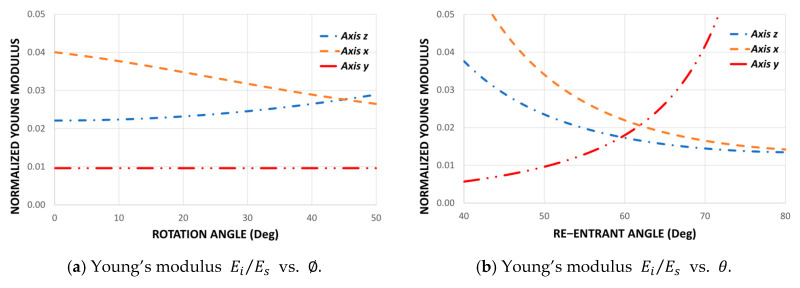
Normalized Young’s modulus $E_{i}/E_{s}$ for different values of the re-entrant angle θ and for different values of the rotation angle ∅. Considering H=13.6 mm, L=5.9 mm, t=1.7 mm, θ=50° and $\emptyset =\emptyset_{1}=\emptyset_{2}=22.5{}^{\circ}$.

This chart could be used as a quick design guide to select the geometric parameters according to the mechanical requirements. It is important to be able to control Poisson’s ratio since it has been shown experimentally that the more negative this property is, the higher the shear strength [38]. Similarly, we can plot the average Young’s modulus in its three orthogonal directions as a function of the re-entrant angle θ and the angle of rotation ∅ since equations (4), (7), and (11) depend on the Young’s modulus $E_{s}$ of the material. However, to show only the influence of topology on the mechanical properties, it can be expressed as a normalized modulus $E_{i}/E_{s}$ for each direction, as shown in Figure 4.

It can be seen from the curves shown in Figure 4 that the rotation angle ∅ does not affect Young’s modulus $E_{y}$ while $E_{x},$ and $E_{z}$ are dependent on ∅. This is explained by the strong dependence of these modules on the design parameters $\theta^{\prime }$, $\theta^{\prime \prime }$, $L^{\prime }$ and $L^{\prime \prime }$ derived by the rotation of re-entrant elements. This also produces a big difference between $E_{x},$ and $E_{z}$, resulting in a new orthotropic auxetic structure.

#### 2.1.5. Orthotropy Quantification

To quantify the degree of orthotropy of the proposed structure, the coefficient presented in this study [66] will be applied. Mechanical orthotropy $I_{Mechanical}$ can be quantified by the ratio difference in Young’s modulus for each direction of work.

(12) $$I_{Direction}=\frac{\sqrt{{\left(E_{x}-E_{Direction}\right)}^{2}+{\left(E_{z}-E_{Direction}\right)}^{2}+{\left(E_{y}-E_{Direction}\right)}^{2}}}{E_{Direction}}$$

(13) $$I_{Mechanical}=\frac{I_{x}+I_{z}+I_{y}}{3}$$

where $I_{Direction}$ represents the orthotropic coefficient measured in a specific direction. $E_{x}$, $E_{z}$ and $E_{y}$ represent the mechanical values for Young’s modulus in each working direction. $I_{Mechanical}$ is the orthotropic coefficient that specifies the deviation of Young’s modulus from its transverse directions.

### 2.2. Experimental Procedure

**Figure 5:**
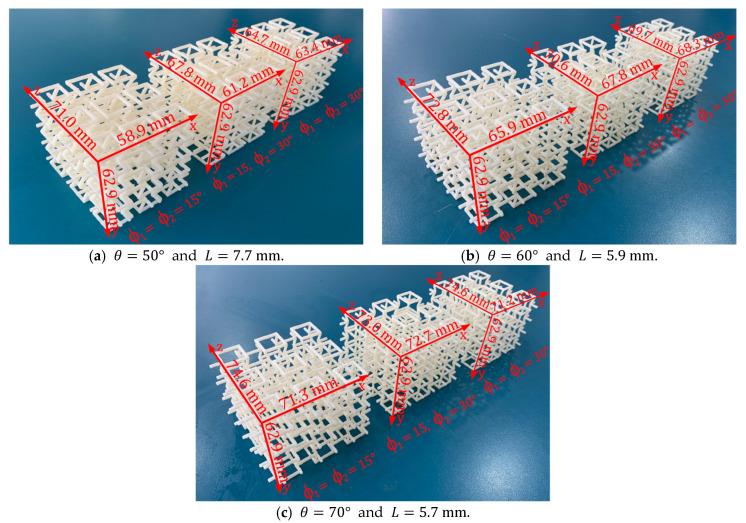
Macrostructures made up of 36-unit cells (3×3×4) manufactured through fused filament fabrication. H=13.6 mm and t=1.70 mm are considered for all cases.

The experimental samples were built using a Stratasys uPrint SE 3D printe, equipped with fused deposition modeling (FDM) technology. The raw material of the filament is ABSplus. The Young’s modulus of the raw material processed by FDM denoted as $E_{s}$, has been characterized in previous works for each manufacturing direction [64,65]. As is known, due to the layer-by-layer manufacturing process, an intrinsic anisotropy is introduced into the structure. Therefore, to reduce the isotropic effect of the FDM process, all the specimens are manufactured on the xz plane, while the y axis is the printing direction. In this way, the design parameter *ϕ* controls the anisotropy of the structure. Three groups of macrostructures of 36-unit cells were manufactured (3×3×4) under different values for re-entrant angle θ={50°;60°;70°}. In addition, within each group, three samples were manufactured with different values for the angle of rotation ∅={15°;22.5°;30°}. It is worth mentioning that the structures with a rotation angle of 15° and 30° are symmetric, that is, the angles $\emptyset_{1}=\emptyset_{2}$ (Figure 1a). On the other hand, the structure with a rotation angle of 22.5° is asymmetric, where $\emptyset_{1}=15{}^{\circ}$ and $\emptyset_{2}=30{}^{\circ}$, so average mechanical behavior is expected. The dimensions for each macrostructure are shown in Figure 5.

**Figure 6:**
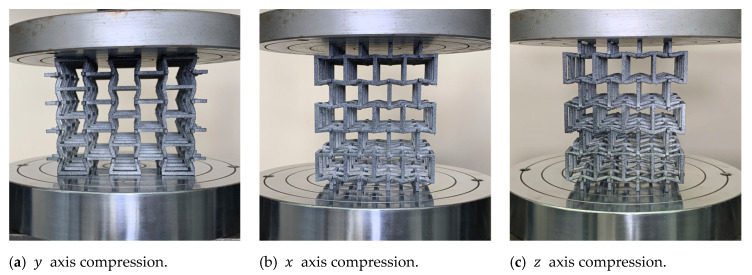
Quasi-static compression tests on each proposed macrostructure in its three orthogonal directions.

**Figure 7:**
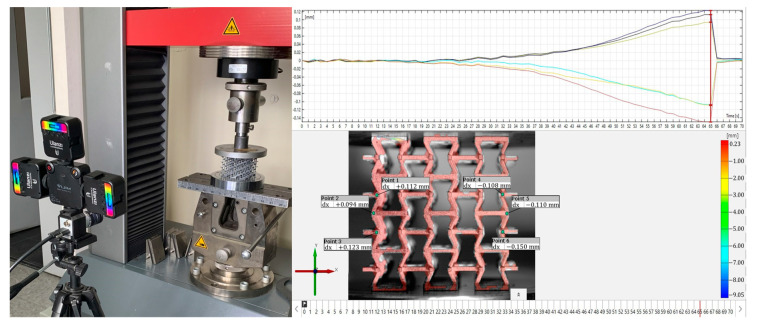
Experimental setup to measure the transverse deformations of structures using an image correlation system through a Basler ace *Aca*1300−200 *uc* camera.

To validate the design of this new structure, quasi-static compression tests were carried out at a constant speed of 1 mm/min. Each of the samples was compressed in its three orthogonal directions within the elastic range to obtain Young’s modulus and Poisson’s ratio for each direction. Note that the methodology for testing at a constant strain rate was not applied here as the plastic range is not in the scope of this work [67]. Each sample was subjected to compression in the directions x, y, and z within the elastic range, as shown in Figure 6. During the test, the test machine automatically recorded the value of force and displacement along the compression direction. Poisson’s ratio for each sample was obtained by stopping the machine and measuring the sample size in the transverse directions using an image correlation system through a camera Basler ace model *acA*1300−200 *uc*. The digital image correlation technique was applied to measure Poisson’s ratio. The technique has been proven to provide an accurate measurement of displacement and strains for small deformations [58,68]. On each sample, the deformations of three different points were analyzed to calculate an average value and improve the precision of the results. The experimental development setup is shown in Figure 7.

**Figure 8:**
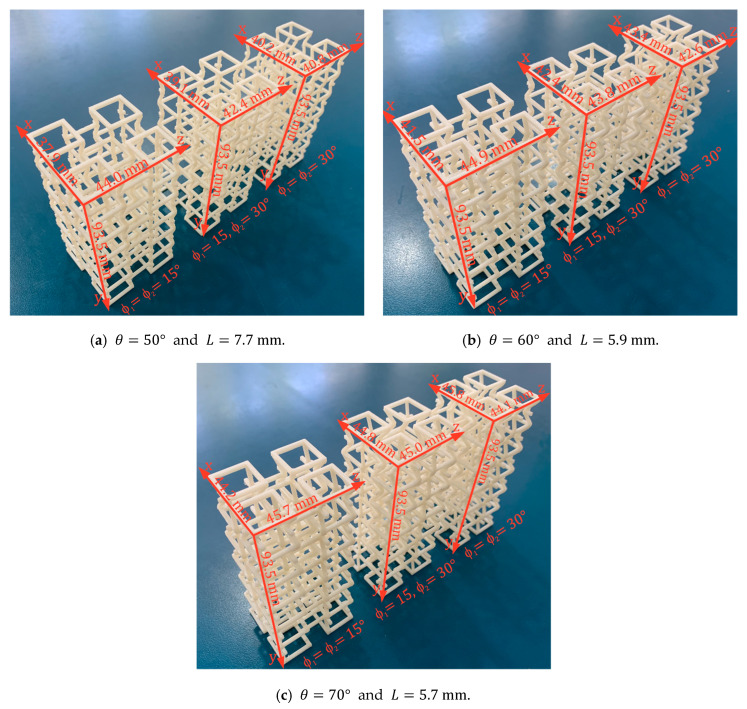
Beams made up of 24-unit cells (2×2×6) manufactured through fused filament fabrication. Considering H=13.6 mm and t=1.7 mm for all cases.

Additionally, three-point bending experiments were carried out at a speed of 1 mm/min. To compare the bending stiffness of each structure, the experiments were carried out with a distance between supports equivalent to the length of 4-unit cells. To study the mechanical behavior that this new structure can experience, three groups of macrostructures of 24-unit cells were manufactured (2×2×6) under different values for re-entrant angle θ={50°;60°;70°}. Using the same geometric configurations as above, within each group, three samples were manufactured with different values for the angle of rotation ∅={15°;22.5°;30°}. The dimensions for each macrostructure are shown in Figure 8.

**Figure 9:**
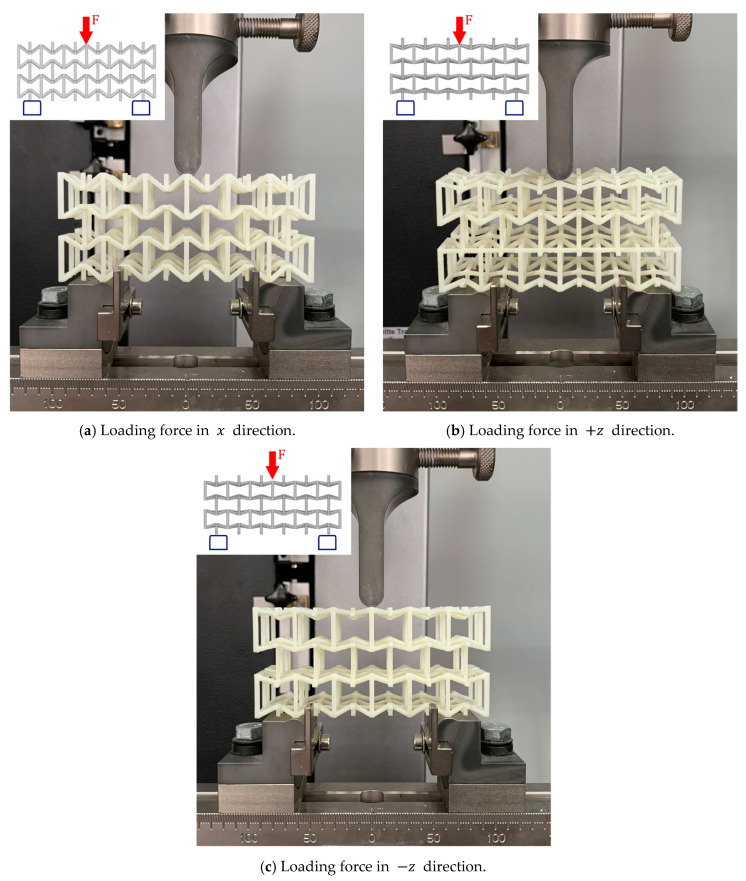
Quasi-static bending tests on the proposed macrostructure in the x, +z, and −z direction.

Each sample was loaded in the *x* and *z* directions. However, the structures that have a rotation angle $\emptyset_{1}=15{}^{\circ}$ and $\emptyset_{2}=30{}^{\circ}$ were tested in the positive direction (+z), and they were also rotated by 180° to be tested in the negative direction (−z), as shown in Figure 9. Due to the asymmetry that this structure possesses, it is expected that it will experience a different mechanical behavior in these two load directions. During all experiments, the testing machine automatically records the applied force and vertical displacement produced.

## 3. Results and Discussion

**Figure 10:**
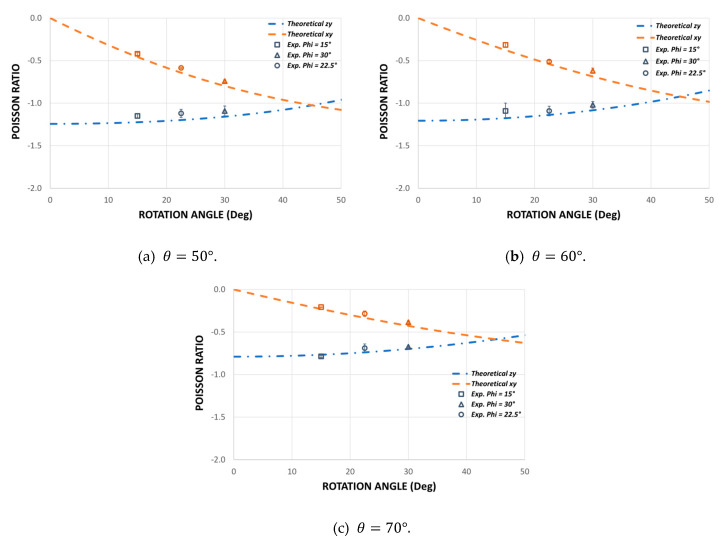
Comparison of the theoretical Poisson’s ratio vs. the experimental Poisson’s ratio for each macrostructure for different values of the re-entrant angle θ={50°;60°;70°} and for different values of the design parameter ∅.

**Figure 11:**
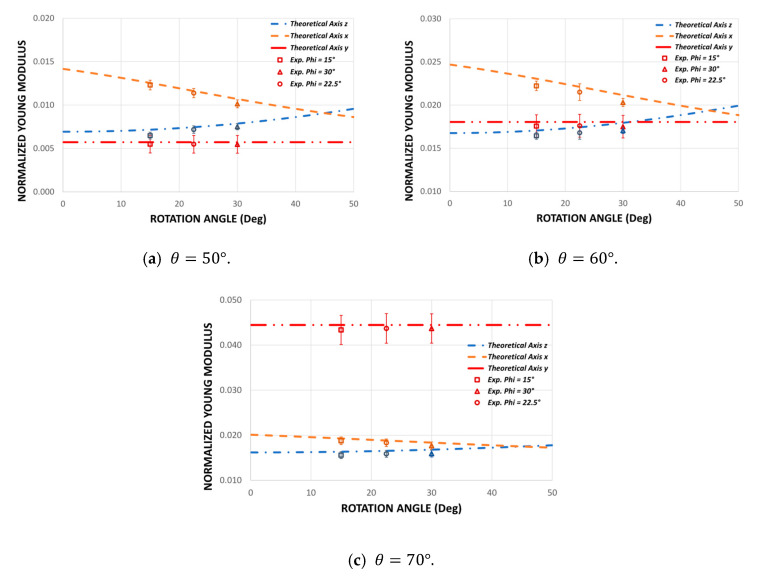
Comparison of the theoretical Young’s modulus $E_{i}/E_{s}$ vs. the experimental Young’s modulus for different values of the re-entrant angle θ={50°;60°;70°} and for different values of the design parameter ∅.

To validate the theoretical design of this new auxetic structure, 27 different experiments were carried out to determine Poisson’s ratio and Young’s modulus in the three orthogonal directions. The experimental results obtained show a good consistency to make a good comparison with the theoretical model. Care was taken at all times to end the experiments with very small strain values to avoid permanent damage. The experimental values are shown in Figure 10 and Figure 11.

### 3.1. Poisson’s Ratio

To study the mechanical behavior of this new auxetic configuration, nine combinations are made for the Poisson’s ratio: three groups for different values of the re-entrant angle θ and three groups for the angle of rotation ∅. The theoretical and experimental results for Poisson’s ratio are shown in Figure 10. In general, there is good agreement between the theoretical prediction and the experimental results for all samples. Although some results for Poisson’s ratio differ slightly from the theoretical values, the trend of the auxetic behavior of each sample agrees well with the theoretical predictions, and the model also manages to capture the effect produced by the new design parameter ∅. Due to the asymmetry of the cell shown in Figure 1a, Poisson’s ratio $v_{zy}$ becomes more negative the higher the value of ∅, whereas Poisson’s ratio *v_xy_* becomes less negative the greater the value of ∅. Thus, for an angle of rotation ∅=45° we have a symmetric structure, and therefore Poisson’s ratios $v_{xy}$ and $v_{zy}$ should be equal. According to the results obtained, it is noted that while the angle of rotation increases ∅, the difference between $v_{xy}$ and $v_{zy}$ decreases. On the other hand, as the objective of this research is to design a cell with different elastic properties in its three orthogonal directions, hence the importance of Poisson’s ratio being $v_{xy}\neq v_{zy}\neq -1$. This avoids equality between transverse deformation and longitudinal deformation in each compression direction.

The experimental results make it possible to validate the theoretical design through Equations (9) and (10) while also demonstrating that the orthotropic behavior of this new auxetic cell can be manipulated by the design parameter ∅. It is important to note that the magnitude of Poisson’s ratio can be manipulated through the geometric parameters and also through the rotation of the re-entrant elements (Figure 1). These results validate the theoretical design and allow a quick manipulation of the mechanical behavior of the structure through its geometric parameters. According to [53], if control over Poisson’s ratio is achieved, it is also possible to achieve control over Young’s modulus over a wide range.

### 3.2. Young’s Modulus

Figure 11 shows the experimental results of the structures subjected to compression for three values of the re-entrant angle θ={50°;60°;70°} and for three values of the rotation angle ∅={15°;22.5°;30°}. The experimental results show that there are differences in the elastic moduli for each compression direction. It can be seen that the modulus of elasticity in the direction y remains constant as a function of the angle of rotation ∅. When compressing the structure on the plane xz (Figure 1a), the bending of the re-entrant struts does not depend on the angle of rotation ∅. On the other hand, the modulus of elasticity in the direction x decreases as the angle of rotation ∅ increases, while the elastic modulus in the z direction increases. The theoretical model manages to capture very well the dependence of the elastic moduli according to the rotation angle ∅. Unlike the design proposed in previous works [64], this design allows greater orthotropy, since the smaller the rotation angle ∅, the greater the difference between the $E_{x}\;$and $E_{z}$ modules. In addition, the structure composed of two different rotation angles $\emptyset_{1}=15{}^{\circ}$ and $\emptyset_{2}=30{}^{\circ}$ maintains an average mechanical behavior in all cases. The trend of the mechanical behavior agrees well with the theoretical predictions, thereby validating Equations (4), (7), and (11). In this way, the theoretical development of this work is a good approximation for the design of this new auxetic structure, which would allow this model to be used as a quick guide for selecting suitable geometric parameters according to the application. On the other hand, it is important to note that a possible cause of the differences between the theoretical predictions and the experiments may be the variability in the size of the struts given that the surface built by the FDM processes is not smooth due to the well-known step effect that occurs in re-entrant struts [69].

Generally, these types of structures made from polymers are not stiff enough for structural applications. However, it is expected that the stiffness can be significantly increased by a good selection of geometric parameters. Thanks to mathematical simplicity, the unit cell design could easily be incorporated into the design of a multicellular structure. Furthermore, this design approach could be applied in future studies to explore mechanical behavior under energy absorption for each work direction. 

#### Orthotropy Quantification

**Figure 12:**
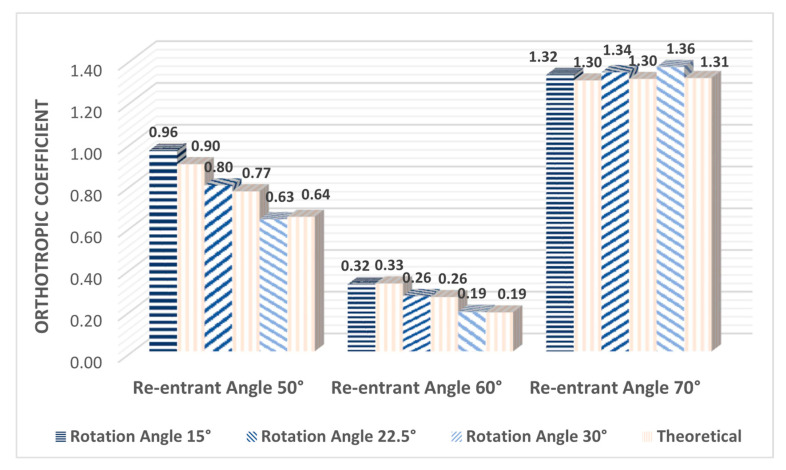
Mechanical orthotropy coefficients for all samples constructed with different values for the re-entrant angle θ={50°;60°;70°} and different values for the angle of rotation ∅.

Figure 12 shows the calculation of the orthotropy coefficient for each experimental sample, according to Equations (12) and (13). This coefficient helps to quantify the level of orthotropy of each configuration; the larger the value of $I_{Mechanical}$, the greater the variation that exists between the elastic moduli. As it was observed in the previous section, the new design parameter ∅ produces an inversely proportional relationship between Young’s moduli $E_{x}$ and $E_{z}$, the smaller the Angle of rotation ∅, the greater the difference between these elastic moduli. Therefore, it was expected that for structures with a rotation angle ∅=15°, a greater orthotropy would be obtained. This behavior is clearly described by the orthotropy coefficient. It can be seen in Figure 12 that for structures that have a re-entrant angle of 50° and 60°, the level of orthotropy increases the smaller the rotation angle ∅. In addition, it can be observed numerically that the structures with a re-entrant angle of 50° reach higher levels of orthotropy than the structures with a re-entrant angle of 60°, as can be seen in the graphs of the previous section. However, it can be seen that the structures that have a re-entrant angle of 70° have a higher orthotropic coefficient, and this remains practically constant as a function of the rotation angle ∅. This occurs due to the low inclination of the re-entrant struts; the structure is stiffer in the direction of the axis y, therefore, the Young’s modulus $E_{y}$ is much greater than the modules $E_{x}$ and $E_{z}$. In addition, Young's modulus $E_{x}$ and $E_{z}$ maintain very little difference between them and vary slightly as a function of the rotation angle ∅. This coefficient supports our observations and helps to establish a comparison criterion to evaluate the degree of orthotropy between the different auxetic configurations. In addition, it helps to validate the theoretical design since it can be seen that in all cases the theoretical coefficient maintains a very good agreement with the coefficient calculated with the experimental data.

### 3.3. Flexural Stiffness

**Figure 13:**
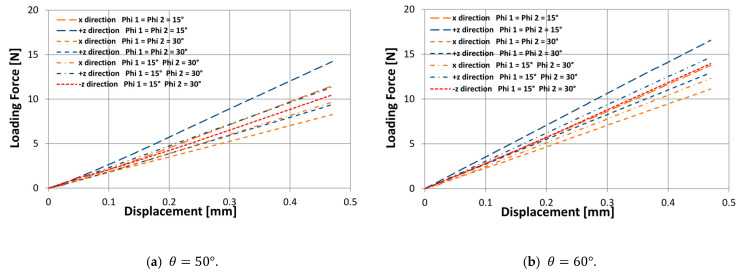

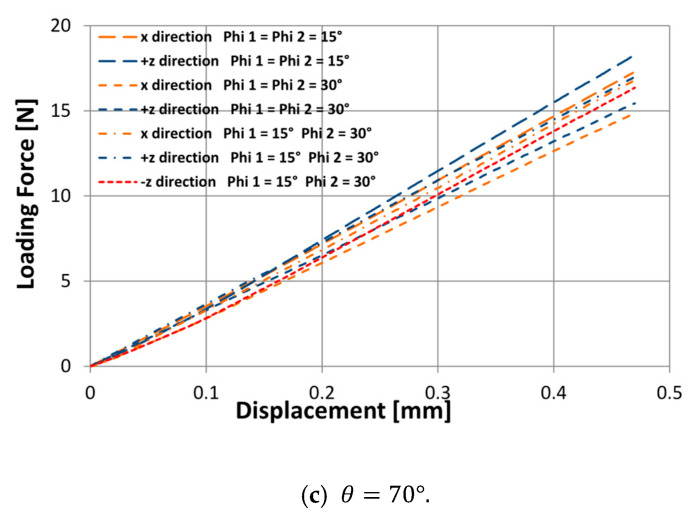
Bending tests performed on the macrostructures of 2×2×6 cells under load force in the x, +z, and −z directions.

According to the results obtained experimentally, the proposed design allows an orthotropic macrostructure to be achieved under compressive stresses; therefore, to evaluate the mechanical behavior under bending stresses, 21 three-point bending experiments were carried out. Figure 13 shows the experimental results of the macrostructures subjected to bending for three values of θ={50°;60°;70°}, and for three values of ∅={15°;22.5°;30°}.

The results show that the stiffness behavior under the x load direction is less than the stiffness under the +z load direction, this trend is observed in all cases, and this difference in stiffness behavior increases the smaller the angle of rotation ∅ and the smaller the re-entrant angle θ. On the other hand, structures that have a rotation angle $\emptyset_{1}=15{}^{\circ}$ and $\emptyset_{2}=30{}^{\circ}$, due to asymmetry, can also experience a different stiffness behavior under the −z load direction. It can be seen that the structures are slightly stiffer under the +z load direction, and their difference with the −z load direction increases while the re-entrant angle θ is smaller. Unlike previous works [64], this new design parameter ∅ obtains a greater difference in the stiffness behavior according to the load direction. Thus, this newly proposed design transmits to the macrostructure a different stiffness behavior in up to three different load directions. However, it is important to validate this hypothesis with SLS or DED technologies [70,71] since the layer-by-layer interface of FDM processes provides a fragile bonding that limits the plastic behavior of the structure. Moreover, the final aim of this orthotropic cell is to apply it to the manufacture of a prosthetic ankle.

## 4. Conclusions

This study presents the design of a re-entrant auxetic structure with different elastic properties in its three orthogonal axes. Using the classical Timoshenko beam theory to model the bending of its re-entrant elements, an asymmetric three-dimensional configuration is established based on a new design parameter ∅. This new design parameter produces an internal rotation of the re-entrant elements of the cell, which allows to manipulate Poisson’s ratio and Young’s Modulus. The mechanical properties of each structure were determined experimentally in the three orthogonal directions. Timoshenko’s simplified theoretical model has been shown to predict with great accuracy the mechanical properties of this new orthotropic structure.

To validate the design of this orthotropic structure, nine macrostructures were fabricated through a fused deposition molding system using ABSplus. The structures were designed with different values for the rotation angle ∅ and for the re-entrant angle θ. Each sample was subjected to quasi-static compression experiments in its three orthogonal directions to determine Poisson’s ratio and Young’s modulus. The proposed auxetic design, the mechanical analysis, and experimental results obtained in this work make it possible to draw the new design parameter ∅, which can manipulate Poisson’s ratio and Young’s modulus up to 25% and 39% of the original values (for a ∅=45°), respectively. Furthermore, ∅ generates an inversely proportional relationship between Young’s moduli *x* and z since with the greater angle of rotation, the Young’s Modulus $E_{x}\;$decreases, while the Young’s Modulus $E_{y}$ increases. This behavior could be described by the orthotropy coefficient that allowed us to quantify the deviation of Young's modulus of each structure. On the other hand, the simplified Timoshenko model is shown to predict the mechanical properties with a maximum error of 9.5% for the validation structures. This allows the theory to be used as a rapid design tool for real engineering applications. In addition, the lattice structure subjected to bending shows a differentiated response of up to 20.1% of bending stiffness in the load directions x and z. This result permits the application in the design of complex structures that require a differentiated behavior according to the direction of work from the simple analysis of the unit cell. In addition, the results of this research were carried out with specific parameters within the elastic zone of the structure. Future research will focus on the study of the plastic behavior of the proposed structure and validation using samples manufactured with selective laser melting technology. Furthermore, the impact energy absorption capacity in its three orthogonal directions will be studied, and this orthotropic cell will also be used in the manufacture of a prosthetic ankle.
